# Thai Rat-Tailed Radish Prevents Hepatocarcinogenesis in Rats by Blocking Mutagenicity, Inducing Hepatic Phase II Enzyme, and Decreasing Hepatic Pro-Inflammatory Cytokine Gene Expression

**DOI:** 10.3390/cancers15061906

**Published:** 2023-03-22

**Authors:** Piman Pocasap, Natthida Weerapreeyakul, Rawiwan Wongpoomchai

**Affiliations:** 1 Department of Pharmacology, Faculty of Medicine, Khon Kaen University, Khon Kaen 40002, Thailand; 2 Human High Performance and Health Promotion Research Institute, Khon Kaen University, Khon Kaen 40002, Thailand; 3 Division of Pharmaceutical Chemistry, Faculty of Pharmaceutical Sciences, Khon Kaen University, Khon Kaen 40002, Thailand; 4 Department of Biochemistry, Faculty of Medicine, Chiang Mai University, Chiang Mai 50200, Thailand

**Keywords:** *Raphanus sativus* L. var. *caudatus* Alef, hepatocarcinogenesis, diethylnitrosamine, rat, immunohistochemistry

## Abstract

**Simple Summary:**

Our previous studies have reported the anticancer activity of *Raphanus sativus* L. var. *caudatus* Alef (RS) in many cancer cells, but only in vitro. The anticancer effects of RS were, therefore, investigated in rats with early-stage liver cancer. RS effectively reduced the overexpression of GST-P positive foci and apoptotic cells in the rats injected with DEN (a carcinogen) during the development of early-stage of cancer. The major finding from this study highlights the chemopreventive activity of RS extract given orally in the initial stage of hepatocarcinogenesis in vivo by (1) inhibiting carcinogenic activities, (2) increasing phase II metabolism, and by (3) lower inflammation. The attributed compounds to these activities could be polyphenols and isothiocyanates, mainly sulforaphene. The results confirm sufficient oral bioavailability, with no detected toxicity, and thus support the use of RS as a health-promoting plant and its possible further study and use in humans.

**Abstract:**

*Raphanus sativus* L. var. *caudatus* Alef (RS) is an indigenous Thai plant with nutritional and medicinal values such as anticancer activity, but only in vitro. The chemopreventive effects of RS were, therefore, investigated in the initial stage of hepatocarcinogenesis in rats. Diethylnitrosamine (DEN), a carcinogen, was intraperitoneally injected into rats to induce liver cancer. Along with the DEN injection, either aqueous (RS-H_2_O) or dichloromethane (RS-DCM) extract was administered orally. Immunohistochemistry was used to detect glutathione *S*-transferase placental (GST-P) positive foci and apoptotic cells in rat livers as indicators of initial-stage carcinogenesis. The underlying mechanisms of chemoprevention were investigated with (a) antimutagenic activity, (b) hepatic phase II enzyme induction, and (c) hepatic pro-inflammatory cytokine gene expression. The results showed that RS-DCM was more potent than RS-H_2_O in decreasing GST-P positive foci and apoptotic cells induced by DEN. The mechanisms of RS-DCM (phenolics and sulforaphene contents) against liver carcinogenesis (1) block the activity of carcinogens; (2) elevate phase II detoxifying enzymes; and (3) suppress the pro-inflammatory gene expression. RS-H_2_O (phenolics contents), in contrast, only decreases pro-inflammatory gene expression. In conclusion, the RS extract consisting of phenolics and isothiocyanates exerted significant chemopreventive activity against DEN-induced liver carcinogenesis.

## 1. Introduction

Brassicaceae (or Cruciferae) is an economically important family consumed worldwide. In addition to nutritional enrichment, Brassica’s phytochemical profiles make the plant family an ideal natural source for health promotion. The phytochemicals, including phenolics, carotenes, minerals, and isothiocyanates (ITCs), are considered health-benefiting constituents. Many studies have demonstrated the potential of these plants against a wide range of complications such as inflammation, infection, diabetes, and, especially, cancer [1]. Epidemiological studies have indicated that the intake of Brassica vegetables is associated with a lower risk of many types of cancer [2]. The study of plants from the Brassica family is, thus, of interest to elucidate and facilitate its use against cancer.

As one of the leading causes of death worldwide, cancer causes tremendous global health problems. In Asia, there is a declining trend in cancer mortality, but the morbidity rate is still increasing, especially among the younger population [3]. In terms of virulence, hepatocellular carcinoma is recognized as one of the most pernicious cancers—ranking as the second cause of cancer death in the Southeast Asia region, where morbidity and mortality incidence is twice the global rate [4]. This is due to the refractory nature of cancer and the lack of awareness due to no specific early signs and symptoms, leading to detection in the late stage. Hence, the countermeasures against liver cancer should not rely only on screening and treatment interventions but also prevention.

The chemopreventive properties of Brassica plants have been demonstrated epidemiologically. One of the crops within the Brassica family displaying promising activities and a distinct chemical profile against cancer is *Raphanus sativus* L. var. *caudatus* Alef (RS, also known as Thai rat-tailed radish). RS is widely distributed in South and Southeast Asia as an indigenous plant and has been used locally as an ingredient in traditional cuisine and medicines [5]. The anticancer activities of RS have been previously reported.

RS aqueous extract consists of several phenolic compounds that contribute to its antiproliferative and antioxidant activity. In contrast, the dichloromethane extract of RS contains ITCs and displays anticancer activities in several types of cancer cell lines [5,6]. The molecular mechanisms of the major ITCs in RS that trigger cancer cell death have also been elucidated [7]. Taken together, RS exhibited anticancer potential and should be further investigated, especially in the in vivo model, to approve its potential against cancer.

This study was, therefore, conducted in vivo using an animal model. The objective of the study is to investigate the preventive effect of RS against liver cancer. The ability of both RS aqueous and dichloromethane extracts to suppress cancer formation in carcinogen-induced initial-stage hepatocarcinogenesis in rats was examined, employing immunohistochemistry procedures. The safety profile of RS extracts was also assessed. The corresponding chemopreventive mechanisms were further investigated both in vitro and in vivo. The outcome of this study could explain the chemopreventive efficacy of RS and provide the fundamental knowledge for further study and clinical application.

## 2. Materials and Methods

### 2.1. Chemicals

2-AA (2-aminoanthracene), AF-2 (2-(2-furyl)-3-(5-nitro-2-furyl)-acrylamide), and MeIQ (2-amino-3, 4 dimethylimidazo[4,5-f]quinoline) were obtained from Wako Pure Chemicals (Osaka, Japan). AFB1 (aflatoxin B1), Bovine serum albumin, diethylnitrosamine, ethanol, DMSO, and NaN_3_ (sodium azide) of analytical grade were purchased from Sigma-Aldrich (St. Louis, MA, USA). Rabbit polyclonal GST placental form (GST-P) antibody was obtained from MBL (Nagoya, Japan). The mouse monoclonal proliferating cell nuclear antigen (PCNA) antibody was from BioLegend (Santiago, CA, USA). EnVision Doublestain system was obtained from Dako (Glostrup, Denmark). The avidin–biotin–horseradish peroxidase complex (ABC) kit was purchased from Vector Laboratories (Burlingame, CA, USA). ApopTaq peroxidase in situ Apoptosis Detection Kit was obtained from Merck (Kenilworth, NJ, USA). Purezol^TM^ Isolation Reagent was from Bio-Rad (Hercules, CA, USA). High-Capacity cDNA Reverse Transcription Kit was purchased from Applied Biosystem^TM^ (Foster City, CA, USA), and SensiFast^TM^ SYBR Lo-ROX Kit was obtained from Bioline (London, UK). Any other chemicals were of analytical grade and were used without any purification.

### 2.2. Plant Materials

RS samples were visually authenticated according to the taxonomy [8] before extraction. RS extracts were prepared as described previously [6]. Briefly, RS pods (harvested at 6–7 weeks) were homogenized with ddH_2_O (1:1, *w/v*) and left for autolysis at 25 °C for 2 h before filtration. The filtrate was then partitioned with dichloromethane (triplicate). The lower-layer dichloromethane and upper-layer water were collected separately. The dichloromethane layer was then dried using a rotary evaporator to yield a final dichloromethane extract (RS-DCM, 0.06% *w/w*), while the aqueous layer was dried using a lyophilizer, yielding 2.87% (*w/w*) of aqueous extract (RS-H_2_O).

### 2.3. Phytochemical Analysis by HPLC

Phytochemical identification in the extracts was performed by comparing the retention time with the standard. The extracts were dissolved in dimethyl sulfoxide. The injection volume was 20 µL. The HPLC analysis was performed with an LC–2030C3D quaternary pump (Shimadzu, Kyoto, Japan), and the stationary phase was a HiQ sil C18W column (4.6 mm × 250 mm, 5 µm) (KYA Technologies Corporation, Tokyo, Japan). The temperature of the column was 38 °C. Mobile phases and elution conditions were performed, as in the previous report [9]. The detection wavelength was 280 nm. The mobile phase comprised solvent A (purified water with acetic acid) and solvent B (acetonitrile) with a flow rate of 0.8 mL/min. The gradient elution was employed from 0 to 5 min with 95–91% solvent A; 5 to 15 min with 91–89% solvent A; 15 to 22 min with 89–85% solvent A; 22 to 30 min with 85–82% solvent A; 30 to 38 min with 82–78% solvent A; 38 to 43 min with 78–20% solvent A; 43 to 46 min with 20–10% solvent A; 46 to 55 min with 10–5% solvent A; 55 to 60 min with 5–95% solvent A; 60 to 65 min with fixed 5% solvent A. Equilibration time was 5 min with 95% solvent A between individual runs. To identify sulforaphene and sulforaphane in the extracts, the mobile phase system was changed to an isocratic 5% THF in ultrapure water (*v/v*) with a flow rate of 1 mL/min for 30 min. The detection wavelength was 210 nm.

### 2.4. Animals and Exposures

Four-week-old male Wistar rats were purchased from the National Laboratory Animal Center, Mahidol University, Nakorn Prathom, Thailand. Rats were acclimatized for 1 week before starting experiments. All rats were housed under controlled conditions (25 ± 1 °C, 50–60% relative humidity, under 12 h light/dark cycle). A basal diet and water were provided ad libitum. The animal protocol was approved by the Animal Ethics Committee of the Faculty of Medicine, Chiang Mai University, Thailand (Approval number: 28/2560) and performed according to institutional guidelines.

### 2.5. Acute Toxicity Test

The acute toxicity test of RS was determined as per a previous report [10] according to the Organization for Economic Co-operation and Development (OECD) guideline 425. Female Wistar rats were randomly divided into 3 groups (5 rats per group). The control was orally given distilled water, while the latter was fed with a single dose (5000 mg/kg BW) of RS-H_2_O. Body weight, signs of toxicity, behavior, and mortality were observed during the first 6 h and every 24 h after administration. On day 14 of the experiment, all rats were euthanized with isoflurane. The internal organs were excised for weighing and gross pathological observations.

### 2.6. Experimental Design

**Figure 1:**
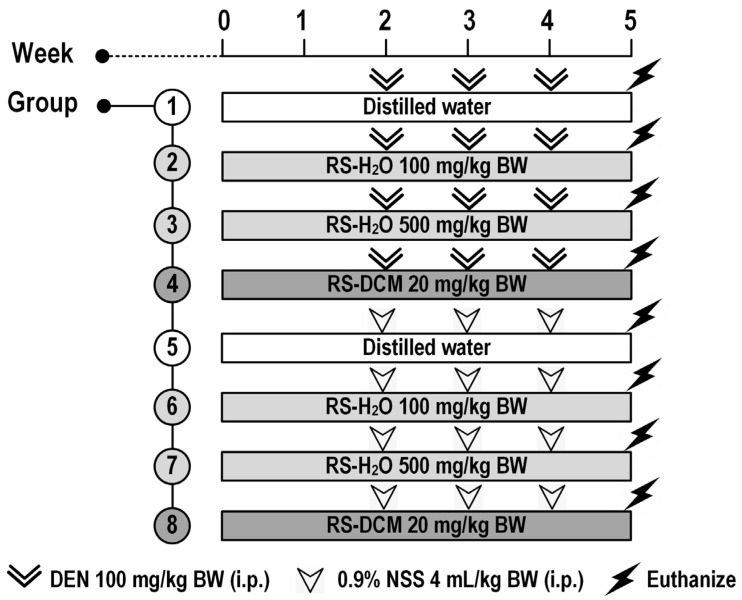
Experimental design to determine the chemopreventive properties of RS in DEN-induced rats. BW: body weight; DEN: diethylnitrosamine; NSS: normal saline solution.

Male Wistar rats were randomly divided into 8 groups (8 rats per group, 64 total) and treated with the samples, as shown in Figure 1. Groups 1 to 4 were injected with DEN (100 mg/kg BW, intraperitoneal) at weeks 2, 3, and 4 to induce early-stage hepatocarcinogenesis. Groups 5 to 8 were injected with NSS (4 mL/kg BW, intraperitoneal) at weeks 2, 3, and 4. Group 1, as a positive control, received distilled water orally, whereas groups 2, 3, and 4 were given RS-H_2_O 100 mg/kg BW, RS-H_2_O 500 mg/kg BW, and RS-DCM 20 mg/kg BW, respectively, from week 0 until the end of the experiment (week 5). Group 5, as a negative control, was given distilled water (with 5% Tween-80) orally, while groups 2, 3, and 4 were fed RS-H_2_O 100 mg/kg BW, RS-H_2_O 500 mg/kg BW, and RS-DCM 20 mg/kg BW, respectively, from week 0 until week 5. Body weight and food/water intake were recorded twice a week. At week 5, all rats were euthanized by exsanguination from the abdominal aorta under isoflurane anesthesia. Whole blood was collected from abdominal veins for alanine transaminase (ALT) activity determination using a commercial kit (Olympus Corp., Tokyo, Japan). The internal organs were excised and weighed. The livers were maintained in 10% formalin, and three serial sections (4 µm thick) were prepared from each specimen. The first section was for histological examination with hematoxylin and eosin staining. The second section was used in immunohistochemistry and molecular analysis, as specified below. The remaining portion was kept at −80 °C for further analysis.

### 2.7. Determination of GST-P Positive Foci

Immunohistochemical staining for GST-P was performed to determine the preneoplastic lesions in rat liver tissues, as previously described [11]. Briefly, the liver sections were deparaffinized and rehydrated with xylene and ethanol. After that, the slides were soaked in H_2_O_2_ (3%) and skimmed milk (1%) to inhibit pseudoperoxidase and inactivate non-specific protein binding, respectively. The samples were incubated with rat anti-GST-P antibody and with secondary antibody (anti-rabbit IgG, ABC kit). Subsequently, the samples were drenched with diaminobenzidine (DAB) and counterstained with hematoxylin. The number and area of GST-P positive foci, with a diameter greater than 0.2 mm, were recorded using the LAS Interactive measurement program (Leica Microsystems CMS GmbH, Mannheim, Germany).

### 2.8. Determination of Apoptotic Cells by TUNEL Assay

To identify apoptotic cells in liver sections, a terminal deoxynucleotide transferase-mediated X-dUTP Nick-End Labeling (TUNEL) assay was performed using an ApopTaq peroxidase in situ kit according to a previous report [12]. The samples were deparaffinized, rehydrated, and pretreated with proteinase and H_2_O_2_ and incubated with equilibrium buffer (5 min) and working-strength terminal deoxynucleotidyl transferase (TdT) enzyme (1 h, 37 °C). After adding stop/wash buffer, the liver sections were treated with an anti-digoxigenin antibody (30 min). The color of TUNEL-positive cells was developed by soaking samples in DAB solution, and methyl green was used to counterstain the specimens. The number of TUNEL-positive hepatocytes was counted at least under 10 fields per liver section under a light microscope.

### 2.9. In vitro Mutagenicity and Antimutagenicity Assay

The mutagenicity of RS was assessed using the Salmonella mutation assay, as previously reported [13]. Briefly, *S*. *Typhimurium* strains TA98 and TA100 were incubated with RS extracts in phosphate buffer, with or without a metabolic activation system (S9 mix). Subsequently, the top agar consisted of 0.05 mM *L*-histidine, and 0.05 mM *D*-biotin was added and poured onto a minimal glucose agar plate. The mixture plates were then incubated for 48 h (37 °C), and the number of histidine-independent revertant colonies was counted. 2-AA and AF-2 were used as standard mutagens (positive control) in the presence or absence of metabolic activation, respectively, while DMSO or distilled water was used as a negative control. S9 mix was prepared from the liver of a male Wistar rat (8–10-week-old) injected intraperitoneally with phenobarbital and 5,6-naphthoflavone. Mutagenicity was displayed as a mutagenic index (MI) calculated from the number of revertant colonies divided by the number of spontaneous revertant colonies. The mutagenicity was classified as a possible mutagen when the MI value was over 2-fold.

Antimutagenicity of RS was performed as per the mutagenicity assay with modification. Without the S9 mix, AF-2 and NaN_3_ were used as standard mutagens in strains TA98 and TA100, respectively. With the S9 mix, AFB1 and MeIQ were used as standard mutagens in strains TA98 and TA100, respectively. The percentage of inhibition of mutagenicity was then calculated following the formula [14]:

$$\%\mathrm{mutagenic}\;\mathrm{inhibition}=\frac{\left(A-B\right)-(C-B)}{\left(A-B\right)}\times 100$$

when A = number of revertants in standard mutagen plates, B = number of spontaneous revertants, and C = number of revertants of test plates.

### 2.10. Determination of Phase II Xenobiotic-Metabolizing Enzymes

Liver microsomal and cytosolic fractions were prepared using the differential centrifugation method according to a previous report [12]. The protein concentration of the sample fractions was determined by the Lowry method.

The activity of phase II metabolizing enzymes was performed as in previous studies [10,15]. UDP-glucuronosyltransferase (UGT) activity was examined by mixing 0.1 M Tris buffer, 4 mM MgCl_2_, 20 mM UDP-glucuronic acid, and 0.5 mM *p*-nitrophenol (PNP) in a microsomal fraction (37 °C, 20 min). The reaction was quenched with 10% TCA in an ice bath and was centrifuged at 10,000× *g* (5 min) prior to alkalinization with 0.5 M NaOH. The absorbance of the mixture was read at 405 nm, and the activity of UGT was calculated using an extinction coefficient of 18 mM^−1^ cm^−1^. The calculated UGT activity values were displayed per mg of protein.

Cytosolic glutathione-*S*-transferase (GST) activity was determined by the reaction with its substrate CDNB (1-chloro-2,4-dinitrobenzoic acid). In brief, the cytosolic fraction was incubated with 0.2 M phosphate buffer, 10 mM GSH, and 10 mM CDNB. The reaction mixture was measured for absorbance at 340 nm. The GST activity was calculated using an extinction coefficient of 9.6 mM^−1^ cm^−1^ and expressed per mg of protein.

The activity of cytosolic NADPH-quinone oxidoreductase (NQO) was examined using DCPIP (2,6-dichlorophenol-indophenol) as an electron acceptor. The rate of DCPIP reduction was measured—in the mixture consisting of 0.025 M Tris-HCl buffer (pH 7.4), 1.0 mg/mL BSA, 1% Tween-20, 150 µM FAD, 30 mM NADPH, and 24 mM DCPIP—an absorbance at 600 nm. The NQO activity was calculated using an extinction coefficient of 2.1 mM^−1^ cm^−1^ and displayed as per mg of protein.

### 2.11. Determination of Pro-Inflammatory Cytokine Gene Expression by Real-Time PCR

Total RNA from the rat liver section was extracted using Purezol^TM^ Isolation Reagent as per the manufacturer’s instruction. cDNA was synthesized according to the manufacturer’s instruction using a High-Capacity cDNA Reverse Transcription Kit. Quantitative real-time PCR was carried out using specific primers (Integrated DNA Technologies, Inc., Singapore), as listed in Table 1. The PCR amplification was performed using SensiFast^TM^ SYBR Lo-ROX Kit. The PCR conditions included initial denaturation at 95 °C (1 min), 40 cycles of denaturation at 95 °C (15 s), annealing at 56–60 °C (15 s), and extension at 72 °C (10 s). The gene expression was normalized with *β*-*actin* and quantified using the 2^−ΔΔct^ method, as previously reported [16].

### 2.12. Statistical Analysis

Data were expressed as means ± SD and were analyzed using SPSS 19.0 for Windows^®^ (SPSS Inc., Chicago, IL, USA). The normality of distribution and homogeneity of variance were analyzed using Shapiro–Wilk tests. The data with a normal distribution, as indicated by *p* > 0.05 according to Shapiro–Wilk tests, were further analyzed using one-way ANOVA (post hoc test: least-significant difference (LSD)). The data with non-normal distribution (*p* ≤ 0.05, Shapiro–Wilk) were analyzed using the non-parametric Mann–Whitney U test. The difference with *p* ≤ 0.05 (parametric LSD’s post hoc test or non-parametric Mann–Whitney U test) was considered statistically significant.

## 3. Results

### 3.1. Phytochemical Identification

The phenolics and isothiocyanates were identified and quantified in the RS extracts by our group previously [9], and the results are presented in Table 2. RS-H_2_O contains several phenolic compounds in a higher amount than RS-DCM. In contrast, RS-DCM comprised a higher isothiocyanate, sulforaphene than RS-H_2_O. The total phenolics in RS-H_2_O compared to RS-DCM were 32.68 and 10.97 mg/g extract, respectively. Vanillic acid was the highest phenolic content in RS-H_2_O extract (26.15 mg/g extract), while *p*-hydroxybenzoic acid was the highest in RS-DCM. The only isothiocyanate compound presented in both was sulforaphene, not sulforaphane. Sulforaphene was mainly detected in RS-DCM (5.11 mg/g extract) (Table 2). Our data indicate that phenolic compounds are enriched phytochemicals in RS-H_2_O, while sulforaphene is the major isothiocyanate in RS-DCM.

### 3.2. Toxicity of RS

To determine the acute toxicity of RS, a single dose of RS-H_2_O (5000 mg/kg) was orally fed to female rats in the treatment groups, in contrast with distilled water in the control group. All the rats in the treatment and control groups survived and showed no visible toxicity signs until the end of the experiment (14 days). There was no difference in water/food consumption or average body and relative vital organ weight between the treatments and control group (Table 3 and Table 4). No internal organ damage was visually detected. Our data suggested that RS extract has no acute toxicity at the treatment dose, and the lethal dose (LD_50_) is supposed to exceed 5000 mg/kg.

**Figure 2:**
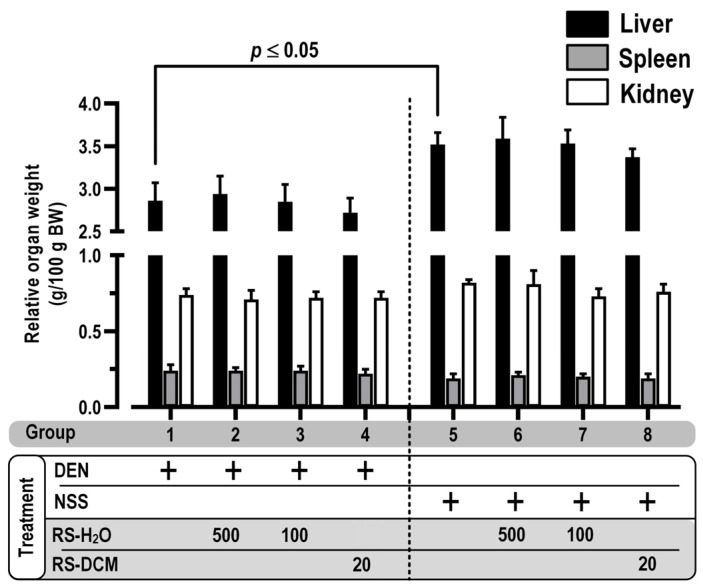
The effect of CP on relative vital organ weight. The values are expressed as mean ± SD. The data were distributed normally (Shapiro–Wilk: *p* > 0.05) and were analyzed for statistical significance using one-way ANOVA with LSD’s post hoc test. RS dosage unit is mg/kg BW.

**Figure 3:**
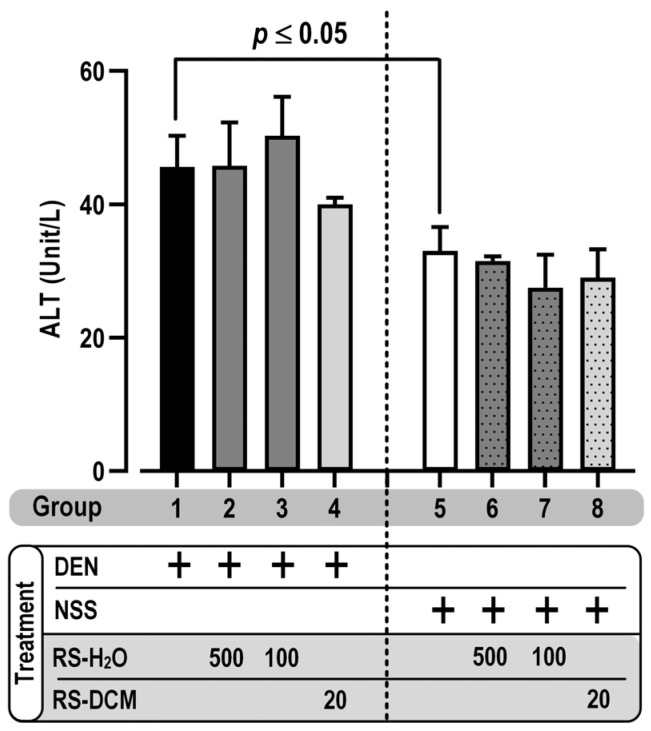
The effect of RS on serum ALT level. The values are expressed as mean ± SD. The data were distributed normally (Shapiro–Wilk: *p* > 0.05) and were analyzed for statistical significance using one-way ANOVA with LSD’s post hoc test. RS dosage unit is mg/kg BW.

The toxicity of RS relevant to the experimental regimen was also observed. Rats were fed daily with RS-DCM (20 mg/kg) or RS-H_2_O (100 and 500 mg/kg) for 5 consecutive days, with or without DEN. The results show no significant effect of RS extracts on average body weight and food/water consumption compared with the positive control (DEN alone, group 1) or negative control (NSS alone, group 5) group (Table 5). For the vital organs, the relative spleen and kidney weight show no significant difference between the groups. In contrast, there is a significant decrease in liver weight between the DEN (group 1–4) and NSS (group 5–8) treatment groups, but no significant difference was observed between groups 1 to 4 and groups 5 to 8 (Figure 2). The data suggested that the liver weight reduction was contributed by DEN, a potent liver carcinogen, not RS. Since carcinogenic liver injury is one of the hepatocarcinogenic processes caused by DEN, the liver inflammatory marker alanine transaminase (ALT) was, therefore, measured to confirm our observations. DEN treatment increased liver inflammation compared with NSS treatment, as indicated by the increasing ALT activity. There was no difference between groups 1 to 4 and groups 5 to 8 (Figure 3). Our results indicated that liver injury was caused by DEN, not by RS. Moreover, the RS extracts have no effect on the observed vital organs during the treatment regimen.

### 3.3. Effect of RS on GST-P Positive Foci

**Figure 4:**
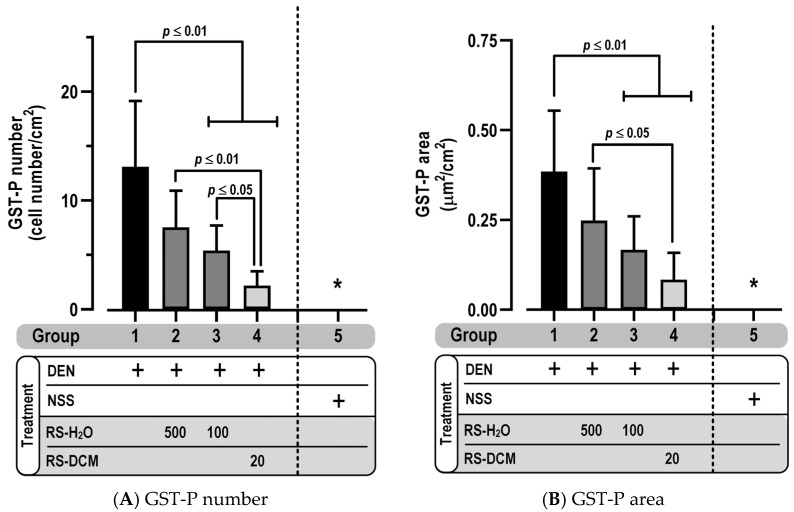
The effect of RS on GST-P positive foci formation as represented by a number (**A**) or area (**B**) per liver area (cm^2^). The values are expressed as mean ± SD. * There are no GST-P positive foci detected in any of the NSS treatment groups (group 5–8). The data display non-normal distribution (Shapiro–Wilk: *p* ≤ 0.05) and were analyzed for statistical significance using the non-parametric Mann–Whitney U test. RS dosage unit is mg/kg BW.

The effect of RS on hepatocarcinogenesis was determined from GST-P foci, a characteristic sign to indicate a preneoplastic lesion in the rat liver. Results showed that GST-P positive foci were observed after DEN administration, whereas NSS treatment found no positive trait. Co-administration of RS-H_2_O (500 mg/kg) or RS-DCM (20 mg/kg) with DEN significantly reduced GST-P positive foci expression (both GST-P number and area) (Figure 4A,B). It was evidence that RS extract potentially attenuated hepatocarcinogenesis.

### 3.4. Effect of RS on Apoptosis Induction

**Figure 5:**
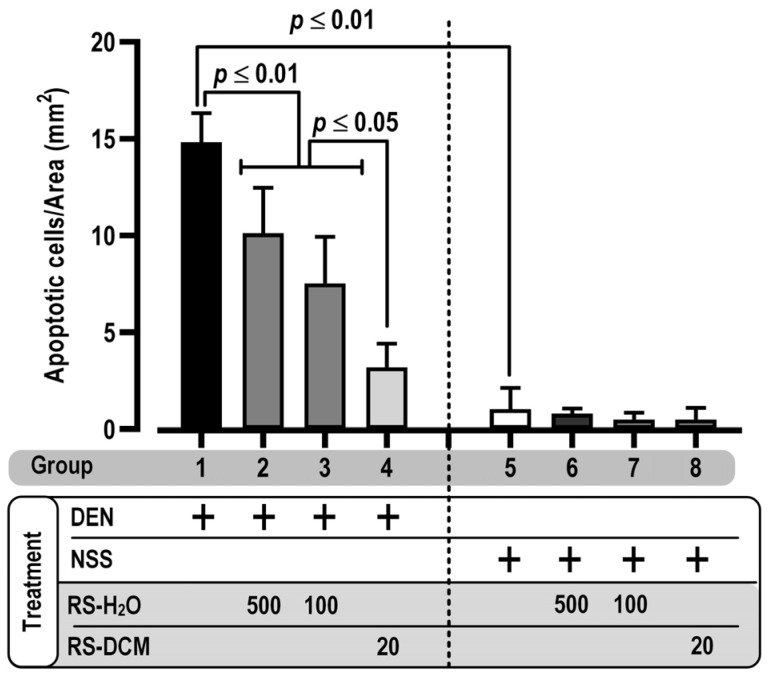
The effect of RS on apoptotic cell expression as indicated using TUNEL assay. The values are expressed as mean ± SD. The data display non-normal distribution (Shapiro–Wilk: *p* ≤ 0.05) and were analyzed for statistical significance using the non-parametric Mann–Whitney U test. RS dosage unit is mg/kg BW.

The effect of RS on hepatocarcinogenesis was determined from apoptotic cell death, a marker of carcinogenesis, using the TUNEL assay. DEN-treated rats displayed a significantly increased number of TUNEL-positive cells (apoptotic cells) in liver tissues than those with NSS treatment. RS treatment (RS-H_2_O and RS-DCM) lessened the apoptosis induced by DEN (Figure 5). Our result indicated the efficacy of RS in suppressing the progression of hepatocarcinogenesis.

### 3.5. In Vitro Mutagenic and Antimutagenic Activity of RS

The potential of RS on mutagenicity was investigated in the Ames test using *S*. *Typhimurium* strain TA98 (frameshift mutation) and TA100 (base-pair substitution) in the presence (+S9) or absence (−S9) of metabolic activation. The mutagenic results showed that both RS-DCM and RS-H_2_O possess no mutagenic activity. No extract at the used concentrations (0.1–5 mg/plate) could increase the number of revertant colonies with a mutagenic index (MI) higher than 2, in contrast with positive controls (AF-2 and 2-AA). RS-DCM at the highest concentration (5 mg/plate) also displayed a killing effect (or cytotoxic) on *S*. *Typhimurium* in both strains. This effect was, however, diminished with metabolic activation (+S9) (Table 6).

**Figure 6:**
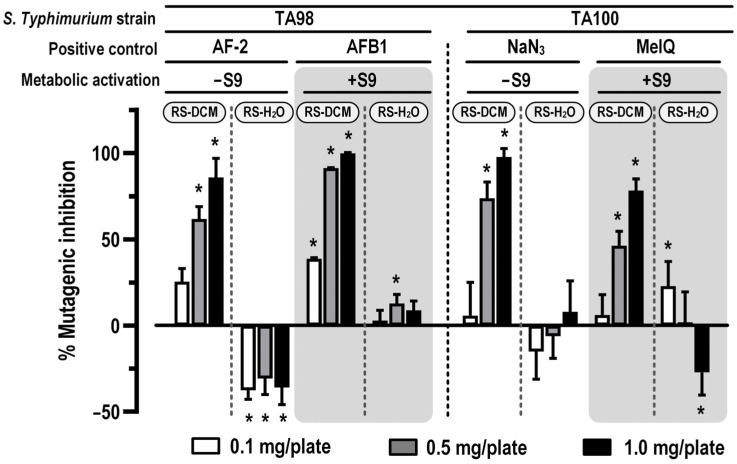
In vitro antimutagenic activity of RS in TA98 and TA100 strains of *S*. *Typhimurium* with non-metabolic (−S9) and metabolic (+S9) activation. The values are expressed as mean ± SD. The data were distributed normally (Shapiro–Wilk: *p* > 0.05) and were analyzed for statistical significance using one-way ANOVA with LSD’s post hoc test. * Significantly different from positive control (set as 0% mutagenic inhibition).

For the antimutagenic activity, the effect of RS extract to reduce mutagenicity was determined in *S*. *Typhimurium* strain TA98 and TA100 by co-treatment with either (1) AF-2 and NaN_3_, as direct mutagens, without metabolic activation or (2) AFB1 and MeIQ, as indirect mutagens, with metabolic activation. To avoid a killing effect, the concentration range of RS extract was determined between 0.1–1 mg/plate (Table 7). Our results indicated that RS-H_2_O has no antimutagenic activity. Additionally, the aqueous extract increased the number of revertant colonies when incubated with both direct and indirect mutagens and possibly acts as co-mutagen. Nevertheless, the co-mutagenic potential of RS-H_2_O is still inconclusive as there is no concentration-dependent correlation observed. In contrast, RS-DCM decreased the revertant colonies (enhancing % mutagenic inhibition) in the co-treatment with both direct and indirect mutagens in a concentration-dependent manner (Figure 6). Therefore, our results indicated that RS-DCM possesses antimutagenic activity.

### 3.6. Effect of RS on Phase II Xenobiotic-Metabolizing Enzymes

**Figure 7:**
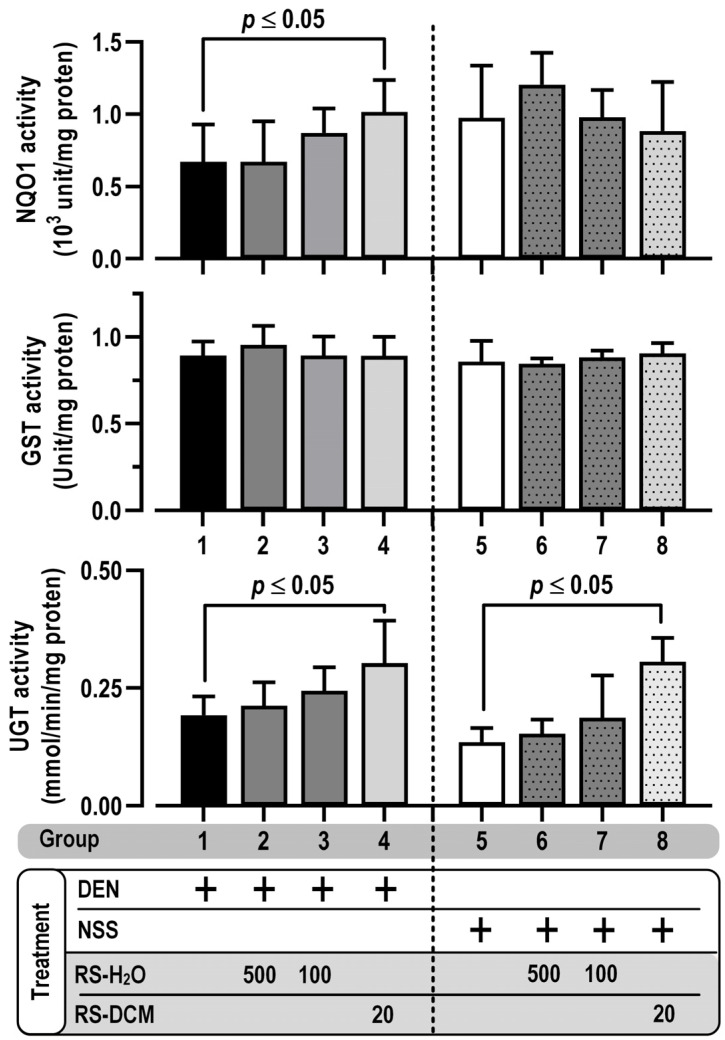
The effect of RS on phase II metabolizing enzymes: UGT, GST, and NQO1. The values are expressed as mean ± SD. The data were distributed normally (Shapiro–Wilk: *p* > 0.05) and were analyzed for statistical significance using one-way ANOVA with LSD’s post hoc test. RS dosage unit is mg/kg BW.

Phase II metabolizing enzymes play a central role in the detoxification of xenobiotics, carcinogens, and reactive oxygen species, attributed to carcinogenesis. Hepatic phase II enzyme activities, including UGT (UDP-glucuronyltransferase), GST (glutathione-*S*-transferase), and NQO1 (NADPH-quinone oxidoreductase) were, thus, determined in this study. Results showed no statistical difference between group 1 (DEN alone) and group 5 (NSS alone) based on the inhibition of phase II enzyme activities. RS-H_2_O treatment did not affect these enzyme activities. On the other hand, UGT and NQO1 activities were increased by RS-DCM co-treatment with DEN. The enhancement of UGT activities after RS-DCM treatment was also observed in the NSS co-treatment group (Figure 7). The data suggested that RS-DCM was more effective than RS-H_2_O in increasing phase II enzyme activities.

### 3.7. Effect of RS on Pro-Inflammatory Cytokine Gene Expression

**Figure 8:**
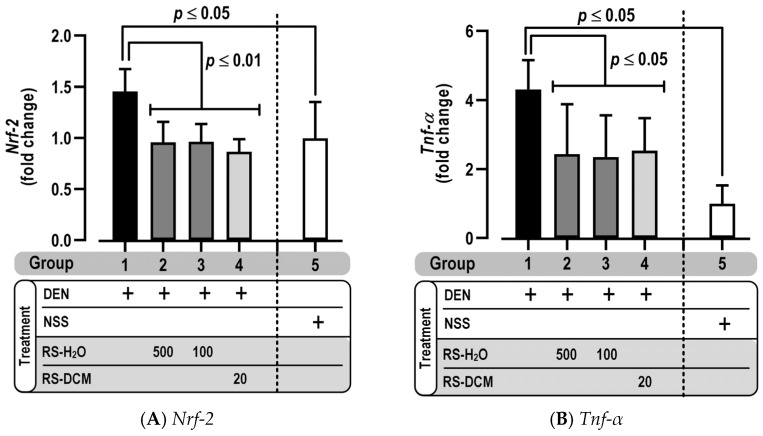
The effect of RS on pro-inflammatory cytokine gene *Nrf*-*2* (**A**) and *Tnf*-*α* (**B**) expression. The values are expressed as mean ± SD. The data display non-normal distribution (Shapiro–Wilk: *p* ≤ 0.05) and were analyzed for statistical significance using the non-parametric Mann–Whitney U test. RS dosage unit is mg/kg BW.

Inflammation is an essential step in carcinogenesis. Pro-inflammatory cytokines such as NRF-2 (nuclear factor erythroid 2–related factor 2) and TNF-α (tumor necrosis factor-α) were, therefore, investigated in our study. DEN treatment increased rat liver inflammation, as indicated by the increased mRNA level of *Nrf*-*2* (Figure 8A) and *Tnf*-*α* (Figure 8B). The overexpression, however, had been downregulated by RS-H_2_O and RS-DCM treatment. These results indicate the ability of RS extract to reduce liver inflammation, an important carcinogenic process.

## 4. Discussion

Genus Raphanus (radish genus) is a rich source of phytochemicals possessing various health-promoting activities. Anthocyanins (i.e., cyanidins and pelargonidins) are commonly found in this genus with health benefits both in vitro and in vivo [17], including cardiovascular protection, anti-inflammatory, and anticancer properties [18]. Raphanus comprises non-flavonoid polyphenols (i.e., phenolic acids, hydroxycinnamates, stilbenes, and tannins) and terpene derivatives (i.e., carotenoids and triterpenoids), which correlated to antioxidant activities in vitro [19,20,21]. Notably, isothiocyanates (ITCs) are the unique constituents in Raphanus and other cruciferous plants, which are responsible for chemoprevention in epidemiological studies [22,23,24].

Our previous studies revealed that RS-DCM consisted of sulforaphene and sulforaphane as the main phytoconstituents [6,8]. The data are in agreement with the present study that isothiocyanate, particularly sulforaphene, is the major constituent. The in vitro data suggested that RS-DCM was capable of inducing HCT116 colon cancer cell death mainly via apoptosis [5]. Moreover, sulforaphene was the predominant active compound [25]. The anticancer mechanisms of sulforaphene, sulforaphane, and other ITCs have been reported to be due to the enhanced intracellular ROS and tubulin depolymerization, as demonstrated using the HepG2 liver cancer cell line [7,26]. In contrast to RS-DCM, RS-H_2_O contains several non-flavonoid polyphenols, including protocatechuic acid, *p*-hydroxybenzoic acid, vanillic acid, caffeic acid, and *p*-coumaric acid (4.2, 1.1, 26.2, 0.6, and 0.7 mg/g extract, respectively) with antioxidant activities. The obtained data suggest that RS extract was a rich source of phytochemicals potentially used for chemotherapeutic purposes. Nonetheless, no previous information indicates the capability of RS against cancer in vivo. The investigation of RS efficacy by employing animal models is, thus, important to support the clinical use of RS.

The rat model of a carcinogen-induced initial stage of hepatocarcinogenesis was established in our study to demonstrate the chemopreventive activity of RS. This was the first time that the acute toxicity of RS was assessed. RS extracts (RS-H_2_O) were safe in rats at a single high dose of 5000 mg/kg orally. The continuous doses for subsequent experiments were the regimen with a reduction ≥10X of the maximum dose (5000 mg/kg) to minimize possible adverse effects. The regimen for RS-DCM was reduced by 250X since our previous study indicated that the RS-DCM extract was more potent than the aqueous extract. During the treatment regimen, RS-DCM (20 mg/kg orally) and RS-H_2_O (100 and 500 mg/kg orally) showed no toxicity as determined by food/water consumption, weight change, and vital organ inspections. Both extracts caused no liver injury, indicated by no alteration in ALT level. Our data indicated that RS extract was safe to administer in rats. The possible confounding factors rendering the RS extracts toxic in rats were, therefore, ruled out.

The initial stage of hepatocarcinogenesis was then initiated by intraperitoneal administration of carcinogen diethylnitrosamine (DEN). In hepatocytes, the carcinogen is subsequently metabolized by cytochrome P450 to form active mutagen and ROS. It was reported that the mutagenic metabolites potentially bind with nucleic acids and trigger DNA mutation, concomitantly with inflammation induced by ROS. Consequently, both DNA mutation and liver inflammation lead to hepatocarcinogenesis [27,28]. In this study, the initial stage of hepatocarcinogenesis was detected by the determination of GST-P positive foci. Since GSTs are a group of hepatic multifunctional proteins participating in the detoxification of toxic and mutagenic agents, the formation of GST-P positive foci depicts the early phase of carcinogenesis due to the enhancement of the detoxification process [29]. Moreover, apoptosis is increased during carcinogenesis as a countermeasure against precancerous cells to prevent excessive cell proliferation [30]. Apoptotic cell death was, therefore, measured to confirm the early stage of cancerous formation.

As indicated by the reduction of GST-P-expressing foci, the early stage of cancerous formation was diminished by both RS-DCM and RS-H_2_O treatments. The data were supported by the TUNEL assay, confirming the efficacy of both RS extracts against the initial stage of hepatocarcinogenesis, as apoptotic cell death was decreased following RS treatments. The results also indicate that the DCM extract is more potent than the aqueous extract. This finding agreed with our previous antiproliferative activity in vitro [6]. ITCs, the predominant active constituents in RS-DCM, might be responsible for the observed chemoprevention in vivo. ITCs prevent carcinogenesis through several mechanisms [31]: (1) blocking the activity of carcinogens, (2) increasing phase II detoxifying enzymes, and (3) inhibiting inflammatory cytokine gene supporting carcinogenesis. The relevant mechanisms regarding ITC activities, therefore, were further investigated.

The ability of RS extracts to block the carcinogenic activity was demonstrated in in vitro antimutagenic tests. In the initiation phase of carcinogenesis, carcinogen (or mutagen) causes irreversible damage to genetic material, resulting in DNA mutation before the promotion and subsequent progression phase occur. Our results showed that RS-DCM possesses antimutagenic activity against direct carcinogens (no metabolic activation required) and indirect carcinogens (metabolic activation required). The data imply that RS-DCM could reduce the activity of carcinogens and lessen the chance of DNA mutation in the initiation phase. ITCs, the active constituents in RS-DCM, possibly play a central role in neutralizing carcinogens since several reports displayed their antimutagenic property. Both sulforaphene and sulforaphane exhibited antimutagenic activity against *S*. *Typhimurium* strain TA98 and TA100 [32]. A previous study reported a positive correlation between daikon (*R*. *sativus* L.) antimutagenic activity and its ITC content [33]. One of the proposed mechanisms of ITCs-neutralizing carcinogenic activity was based on a covalently bound between the isothiocyanates functional group (-N=C=S) and the nitrogen-containing moiety of the heteroaromatic amine of mutagen [34]. In addition, ITCs were reported to compete with procarcinogens to bind with CYP450, interfere with the conversion of procarcinogens to carcinogens, and, finally, decrease carcinogens [31]. Hence, the inhibition of the carcinogens activity of DCM extract could be due to two possible mechanisms, which are (1) direct effect by interacting directly with carcinogens, leading to neutralizing carcinogen reactivity, and (2) indirect effect by reducing carcinogen formation indirectly through competing with procarcinogen transformation via CYP450. For the aqueous extract, there is no antimutagenic activity detected. The concentration of non-flavonoid polyphenol in this extract is possibly insufficient for exerting the observed activity, although the antimutagenicity of these phytochemicals (i.e., vanillic acid) was reported [35].

The ability of RS extract on phase II detoxifying enzymes was further investigated in vivo. Phase II enzymes are important endogenous molecules to prevent carcinogenesis by inactivating carcinogens into a lesser reactive metabolite. In the present study, the detoxifying enzymes (such as UGT and NQO1) were increased in the RS-DCM treatment group. ITCs have been well recognized as potent phase II enzyme inducers [31]. The ITC moiety interacts with thiol residues of KEAP1 (Kelch-like ECH-associated protein 1), leading to the dissociation of KEAP1 from NRF-2. The NRF-2 then translocates freely to the nucleus, where it binds to a transcriptional regulatory element ARE (antioxidant response element) and activates the expression of multiple phase II detoxifying enzymes [36]. The observed enhancement of phase II enzymes after RS-DCM administration is proposed to be due to the presence of ITCs (i.e., sulforaphane and sulforaphane) in the dichloromethane extract. For the RS-H_2_O, there is no phase II enzyme alteration detected after RS-H_2_O treatment. The phenolic content employed in this study (i.e., vanillic acid; 13.1 mg/kg orally) might not be high enough to achieve a significant result as previously reported (vanillic acid ≥ 75 mg/kg orally) [16,37].

The effect of RS extract on pro-inflammatory cytokine gene expressions was examined in vivo since inflammation is critical to carcinogenesis, facilitating tumor growth and survival. RS-DCM and RS-H_2_O treatments decreased *Tnf*-*α* gene expression, whereas *Nrf-2* displayed no alteration after RS treatments. ITCs reportedly suppressed inflammation mainly by inhibition of NF-κB (nuclear factor kappa B), a transcription factor responsible for several pro-inflammatory gene expressions, including *Tnf*-*α*. In normal conditions, NF-κB is retained in an inactive form and sequestered with IκB (inhibitor kappa B). During stress, IκB was phosphorylated and degraded, releasing NF-κB to its active form, which later translocated to the nucleus and acted as a transcriptional activator of many pro-inflammatory genes [38]. ITCs reportedly inhibited IκB phosphorylation, maintaining NF-κB to its inactive form and suppressing the transcriptional activation [39]. Hence, RS-DCM might suppress the expression of *Tnf*-*α*, a pro-inflammatory cytokine gene, by inhibiting NF-κB. Additionally, ITC (sulforaphane) displays no in vivo effect on *Nrf-2* gene expression [40], which is in agreement with our result. For the aqueous extract, the polyphenols might contribute to the suppression of *Tnf*-*α*. For example, vanillic acid (10 mg/kg orally) decreased the expression of *Tnf*-*α* and several pro-inflammatory genes in rats [41], whereas intraperitoneal administration of protocatechuic acid downregulated the pro-inflammatory gene, displaying hepatoprotective effect in rats [42]. The proposed mechanism is related to the upstream regulation of NF-κB [43,44]. Nevertheless, the exact molecular mechanism is yet to be elucidated, and other phytochemicals possibly contributed to the observed result.

Carcinogenesis requires three stages of development, including initiation, promotion, and progression. Carcinogens play the most important role in the initiation stage, triggering DNA mutation, whereas phase II metabolism nullifies the effects. The inflammation then takes part mainly in the promotion and progression stage, sustaining excessive cell proliferation. The coherence of these factors (carcinogens, phase II enzymes, and pro-inflammation) is manifested in several types of cancer [45]. For example, metabolic activation of procarcinogen is an essential step in initiating carcinogen-induced colon carcinogenesis. Phase II detoxifying enzymes then respond to counteract the cellular stress. Subsequently, inflammatory cytokines up-regulate to facilitate cancer growth [46]. Our results show that by (1) inhibiting carcinogenic activities and (2) increasing phase II metabolism, RS extract may dampen the triggering factors in the initiation stage. Moreover, by (3) lower inflammation, RS extract reduces the supportive factors in the promotion and progression stage. Taken together, RS displays chemopreventive properties in many steps of carcinogenesis. In addition, the protective effect could be reached with oral administration reflecting sufficient oral bioavailability, with no detected toxicity. Furthermore, with the efficacy, safety, and bioavailability, our data support the applications of RS as a dietary supplement for chemoprotective purposes.

As of the current knowledge of hepatocarcinogenesis, characteristic inflammation is the crosstalk between carcinogen (i.e., aflatoxin) and non-carcinogen (i.e., viral infection and diabetes) induced hepatocellular carcinoma [47,48,49]. Aberrantly activation of the key signaling pathway Akt/mTOR in liver cancer contributes to the deregulation of the cell cycle, proliferation, cell death, and inflammation [50,51]. Pro-inflammatory cytokine TNF-α is positively associated with overactive Akt/mTOR signaling at both upper and lower downstream levels [47,52]. Our results showed that RS-DCM and RS-H_2_O suppress *Tnf*-*α* expression, implying the potential chemopreventive use of RS not only for the carcinogen but also for non-carcinogen induced tumor formation. Further studies should, therefore, include the investigation of RS on non-carcinogen induced hepatocarcinogenesis in vivo. A pharmacokinetic profile tracking bioactive compounds (i.e., sulforaphene and vanillic acid) in animal models, as well as a more in-depth safety profile, should also be evaluated to extrapolate the use of the RS for a further clinical study

## 5. Conclusions

The data demonstrate the chemopreventive efficacy of RS against initial-stage hepatocarcinogenesis induced by a carcinogen in rats. The dichloromethane extract RS-DCM was more potent than its aqueous counterpart. The proposed mechanism for the RS-DCM (containing sulforaphene, *p*-hydroxybenzoic acid, and other phenolics) was to exert its preventive activity via (1) blocking the activity of carcinogen through both direct and indirect interactions; (2) enhancing phase II detoxifying enzymes; and (3) suppressing the pro-inflammatory cytokine gene expression. In comparison, the water extract RS-H_2_O (mostly contained vanillic acid and other phenolics) could only downregulate the pro-inflammatory gene, and that possibly explains the difference in potency between the two extracts. Aside from therapeutic activity, the safety profile was also considered. In our animal model, oral administration of RS extracts showed no observable toxicity—both acute (single high dose regimen) and subacute toxicity (treatment regimens)—and had no inflammatory potential against hepatocytes. The two extracts displayed no mutagenicity in vitro. Neither extract can trigger and facilitate carcinogenesis in a rat’s liver. Collectively, RS extracts exhibited a favorable safety profile. Furthermore, regarding the treatment regimen, our data demonstrated that oral administration of RS could achieve sufficient bioavailability and yield therapeutic response efficiently. Both RS extracts were a potential natural source attributed to plausible chemopreventive agents for cancer prevention.

## Figures and Tables

**Table 1:** The primer sequences for pro-inflammatory cytokine gene.

Gene		Primer Sequence	Reference
*Nrf-2*	Forward	5′-GCC AGC TGA ACT CCT TAG AC-3′	[10]
	Reverse	5′-GAT TCG TGC ACA GCA GCA-3′	
*Tnf* *-α*	Forward	5′-AAA TGG CCC TCT CAT CAG TCC-3′	[15]
	Reverse	5′-TCT GCT TGG TGG TTT GCT ACG AC-3′	
*β* *-actin*	Forward	5′-ACA GGA TGC AGA AGG AGA TTA C-3′	[15]
	Reverse	5′-AGA GTG AGG CCA GGA TAG A-3′	

**Table 2:** Phytochemicals content of RS extract.

Phytochemicals		Extract (mg/g Extract)	
		RS-H_2_O	RS-DCM
Phenolics			
	Protocatechuic acid	4.21 ± 1.60	2.33 ± 0.03
	*p*-Hydroxybenzoic acid	1.06 ± 0.18	6.01 ± 0.06
	Vanillic acid	26.15 ± 8.97	nd
	Caffeic acid	0.63 ± 0.05	0.43 ± 0.02
	*p*-Coumaric acid	0.66 ± 0.06	nd
	Ferulic acid	nd	2.20 ± 0.01
Isothiocyanates			
	Sulforaphane	nd	nd
	Sulforaphene	0.72 ± 0.02	5.11 ± 0.23

The values are expressed as mean ± SD. nd: not detected.

**Table 3:** Effects of high single dose RS on rat body weight (BW) and food/water consumption.

Treatments	Weight (g)		Consumption (g/rat/day)	
	Initial	Final	Food	Water
DW	172 ± 12	181 ± 14	13 ± 1	25 ± 0
RS-H_2_O (5000 mg/kg BW)	173 ± 16	205 ± 14	14 ± 0	29 ± 3

The values are expressed as mean ± SD.

**Table 4:** Effects of high single dose RS on relative vital organ weight.

Organs		Treatments	
		DW	RS-H_2_O
			(5000 mg/kg BW)
Relative weight (g/100 mg BW)	Liver	3.19 ± 0.15	4.21 ± 0.45
	Spleen	0.20 ± 0.02	0.20 ± 0.03
	Kidney	0.76 ± 0.05	0.85 ± 0.05
	Lung	0.45 ± 0.31	0.43 ± 0.01
	Heart	0.31 ± 0.03	0.30 ± 0.02
	Pancreas	0.38 ± 0.06	0.38 ± 0.06
	Adrenal gland	0.0366 ± 0.0055	0.0402 ± 0.0056
	Ovary	0.0631 ± 0.0118	0.0560 ± 0.0097
	Uterus	0.27 ± 0.08	0.25 ± 0.06

The values are expressed as mean ± SD.

**Table 5:** Effects of RS on rat body weight (BW) and food/water consumption.

Group	Treatment		Weight (g)		Consumption (g/rat/day)	
	Initiator ^#^	Test Compound	Initial	Final	Food	Water
1	DEN	DW	88 ± 17	271 ± 17	21 ± 0	27 ± 9
2	DEN	RS-H_2_O 500 mg/kg	86 ± 18	260 ± 22	21 ± 0	31 ± 10
3	DEN	RS-H_2_O 100 mg/kg	83 ± 16	261 ± 21	21 ± 1	27 ± 5
4	DEN	RS-DCM 20 mg/kg	86 ± 18	271 ± 19	21 ± 2	23 ± 7
5	NSS	DW	80 ± 14	289 ± 20	21 ± 0	31 ± 8
6	NSS	RS-H_2_O 500 mg/kg	89 ± 22	300 ± 19	23 ± 2	27 ± 1
7	NSS	RS-H_2_O 100 mg/kg	86 ± 17	308 ± 24	21 ± 1	30 ± 7
8	NSS	RS-DCM 20 mg/kg	84 ± 20	297 ± 31	20 ± 2	25 ± 8

^#^ DEN and NSS were used as a positive and negative control, respectively, to initiate hepatocarcinogenesis. The values are expressed as mean ± SD.

**Table 6:** In vitro mutagenic activities of RS extract in TA98 and TA100 strains of *S. Typhimurium* with non-metabolic (−S9) and metabolic (+S9) activation.

Treatments		TA98				TA100			
		−S9		+S9		−S9		+S9	
Dose (mg/plate)		Revertant Colonies	MI	Revertant Colonies	MI	Revertant Colonies	MI	Revertant Colonies	MI
Negative control									
DMSO		17.1 ± 3.2		23.4 ± 4.9		109.4 ± 15.9		95.0 ± 12.6	
DW		24.0 ± 3.8		29.4 ± 5.1		106.4 ± 14.6		99.0 ± 15.9	
Positive control									
AF-2	1 × 10^−4^	388.8 ± 38.5 *							
	1 × 10^−5^					427.2 ± 39.9 *			
2-AA	5 × 10^−4^			618.0 ± 52.1 *				1452.0 ± 144.7 *	
RS-H_2_O	0.1	18.8 ± 2.5	0.8	25.9 ± 5.4	0.9	96.2 ± 17.9	0.9	97.3 ± 15.8	1.0
	0.5	21.3 ± 5.6	0.9	27.8 ± 3.9	0.9	101.5 ± 15.5	1.0	111.2 ± 13.8	1.1
	1.0	19.6 ± 2.8	0.8	29.8 ± 5.2	1.0	99.7 ± 21.8	0.9	109.6 ± 12.8	1.1
	5.0	19.0 ± 4.5	0.8	34.1 ± 7.4	1.2	117.1 ± 13.7	1.1	115.3 ± 17.6	1.2
RS-DCM	0.1	18.8 ± 3.2	1.1	23.9 ± 4.8	1.0	106.1 ± 13.1	1.0	93.2 ± 18.7	1.0
	0.5	19.4 ± 8.0	1.1	24.8 ± 2.0	1.1	79.3 ± 16.5	0.7	88.6 ± 13.8	0.9
	1.0	16.6 ± 5.9	1.0	22.1 ± 3.7	0.9	66.2 ± 23.3	0.6	91.7 ± 13.4	1.0
	5.0	5.6 ± 5.9 *	0.3 ^K^	18.1 ± 6.2	0.8	40.3 ± 33.0 *	0.4 ^K^	94.5 ± 21.6	1.0

The values are expressed as mean ± SD. MI: Mutagenicity index. DMSO or DW (50 µL/plate) were the negative control. * Statistically significant compared with negative control in the same column. ^K^: Killing effect (MI ≤ 0.6).

**Table 7:** In vitro antimutagenic activities of RS extract in TA98 and TA100 strains of *S*. *Typhimurium* with non-metabolic (−S9) and metabolic (+S9) activation.

Treatment		Revertant Colonies			
Dose		TA98		TA100	
(mg/plate)		−S9	+S9	−S9	+S9
Negative control					
DMSO		18.7 ± 4.5 *	21.7 ± 3.1 *	91.2 ± 6.9 *	85.3 ± 14.2 *
DW		20.2 ± 4.1 *	32.3 ± 3.2 *	98.6 ± 2.4 *	97.7 ± 16.0 *
Positive control					
AF-2	1 × 10^−4^	291.5 ± 31.4			
AFB1	1 × 10^−5^		1233.3 ± 35.3		
NaN_3_	5 × 10^−4^			370.8 ± 44.9	
MeIQ	5 × 10^−4^				1056.3 ± 65.6
RS-H_2_O	0.1	393.2 ± 14.4 *	1198.7 ± 70.7 *	412.0 ± 43.7	836.7 ± 137.1 *
	0.5	374.8 ± 25.2 *	1079.7 ± 62.4	387.7 ± 34.8	1038.8 ± 170.0
	1.0	389.2 ± 26.8 *	1128.0 ± 6.5 *	349.2 ± 48.7	1317.3 ± 125.9 *
RS-DCM	0.1	222.0 ± 20.3 *	762.7 ± 6.1 *	354.7 ± 53.7	997.8 ± 116.0
	0.5	123.0 ± 19.3 *	127.3 ± 4.0 *	164.5 ± 26.3 *	606.7 ± 80.1 *
	1.0	57.7 ± 30.6 *	24.3 ± 6.5 *	98.0 ± 14.0 *	296.0 ± 65.4 *

The values are expressed as mean ± SD. DMSO or DW (100 µL/plate) were used as negative control. * Statistically significant compared with positive control in the same column.

## Data Availability

The data presented in this study are available in this article.
